# Flavonoids Identified in *Terminalia* spp. Inhibit Gastrointestinal Pathogens and Potentiate Conventional Antibiotics via Efflux Pump Inhibition

**DOI:** 10.3390/molecules30112300

**Published:** 2025-05-23

**Authors:** Muhammad Jawad Zai, Matthew James Cheesman, Ian Edwin Cock

**Affiliations:** 1 Centre for Planetary Health and Food Security, Griffith University, Brisbane, QLD 4111, Australia; muhammadjawad.zai@griffithuni.edu.au; 2 School of Environment and Science, Griffith University, Brisbane, QLD 4111, Australia; 3 School of Pharmacy and Medical Sciences, Griffith University, Southport, QLD 4222, Australia; m.cheesman@griffith.edu.au

**Keywords:** antimicrobial resistance, *Terminalia* spp., gastrointestinal pathogens, combinational therapies, synergy, efflux pump inhibitor

## Abstract

The genus *Terminalia* has a long history of use in traditional medicine to treat various diseases, including bacterial infections. We previously reported a metabolomic analysis using liquid chromatography–mass spectrometry of selected Australian *Terminalia* spp. and highlighted numerous flavonoids that may contribute to the antimicrobial activities of those plants. This study examines the antibacterial activities of fifteen flavonoids found in *Terminalia* spp. against a range of gastrointestinal pathogens using broth dilution assays. Flavonoids were also combined with six different classes of conventional antibiotics to investigate interactions. The efflux pump inhibitory activity of the flavonoid was evaluated using ethidium bromide accumulation and efflux assays. Toxicities were assessed via human dermal fibroblast cell line assays. Fisetin, hispidulin, isoorientin, orientin, rutin, and vitexin showed noteworthy growth inhibitory activity (MIC values 62.5–250 µg/mL). Isoorientin and orientin were most potent against *Bacillus cereus* and *Alcaligenes faecalis,* displaying MIC values of 62.5 µg/mL against both bacteria. All flavonoids except genistein, isorhamnetin, kaempferol, luteolin, taxifolin, and vitexin were nontoxic in human dermal fibroblast (HDF) cell proliferation assays. When individual flavonoids were combined with selected antibiotics, some potentiated the activity of these antibiotics. Two synergistic, eighteen additive and thirty-one non-interactive interactions were observed. The synergistic interactions were all observed in combination with orientin. Notably, orientin exhibited efflux pump inhibitory effects at concentrations from 15.26 µg/mL to 125 µg/mL. The findings reported herein indicate that the selected flavonoids have the potential for addressing bacterial antibiotic resistance and highlight the need for further study.

## 1. Introduction

Antimicrobial resistance (AMR) poses one of the greatest challenges to global human health. The spread and transmission of multi-drug resistant (MDR) pathogens has been facilitated by the rapid rise in human travel and trade over the past few decades [1]. Without sufficient intervention, global deaths due to AMR are expected to exceed those from cancer, reaching 10 million per year by 2050 [2]. Notably, almost all of the pathogenic species linked to AMR spend part of their lifecycle in the gut [1]. The human gastrointestinal (GI) tract hosts a highly organized microbial community capable of transmitting and fostering MDR pathogens, with the gut microbiome housing approximately 10^14^ microorganisms [3]. Enteric bacteria constantly interact with external factors, such as antibiotics and foreign organisms, which contribute to the spread and development of AMR. Enteric bacteria are a major cause of human infections, with the human gut acting as a key conduit for the emergence and spread of MDR strains [4]. Entero-pathogens, if not treated properly, can cause extensive morbidities including bacteraemia, shock, dehydration, and even death [5]. Indeed, diarrheal disease is responsible for more than 1.6 million deaths globally and ranks among the top five leading causes of mortality in children under the age of five [6]. Whilst many of these diseases are regionally endemic, increased globalization has significantly accelerated the spread of MDR pathogens [1].

*Shigella* bacteria are a significant cause of diarrheal illness and food poisoning [7]. In 2016, shigellosis ranked as the second most common cause of diarrheal deaths worldwide, causing over 200,000 fatalities annually, surpassed only by rotavirus [6]. The *Shigella* genus consists of four primary pathogenic species: *Shigella dysenteriae*, *Shigella flexneri*, *Shigella boydii*, and *Shigella sonnei* [8]. *Shigella* infections were previously highly responsive to readily available and affordable antibiotics, including antifolates and β-lactams [8]. However, increasing resistance rates have shifted the preferred treatments for *Shigella* sp. Infections to the use of fluoroquinolones or macrolides, with ceftriaxone as an alternative option [5]. *Salmonella enterica* is another enteropathogenic species belonging to the *Enterobacteriaceae* family. *Salmonella enterica* serovars are categorized into typhoidal and non-typhoidal *Salmonella* and include bacteria that inhabit the digestive tracts of both animals and humans [9]. Non-typhoidal *Salmonella* infections are typically self-limiting [9]. Therefore, antibiotics are generally avoided when a non-typhoidal *Salmonella* infection is identified, although they are still prescribed by some medical practitioners. Unfortunately, the inappropriate use of antibiotics has induced non-typhoidal *Salmonella* to evolve to produce azithromycin and streptomycin-resistant strains [9]. This is particularly concerning since azithromycin is considered a second-line treatment against such infections resistant to other antibiotics [10].

In recent years, traditional medicinal plants have gained considerable attention in the fight against AMR due to their reported antibacterial properties and their potential to enhance conventional antibiotics’ effectiveness [11]. Plants belonging to the genus *Terminalia* have a long history of traditional use for treating various ailments [12]. Many *Terminalia* spp. demonstrate strong antioxidant properties. Indeed, *Terminalia ferdinandiana* Exell. fruit have been reported to have the highest antioxidant activity of any plant worldwide, with ascorbic acid levels exceeding those found in blueberries by over 900 times [13]. Previously, our group recorded the antimicrobial activity of several *Terminalia* spp. including *Terminalia petiolaris* A. Cunn. ex Benth., *Terminalia canescens* DC. Radlk., *Terminalia grandiflora* Benth., *Terminalia muelleri* Benth., *Terminalia microcarpa* Decne., and *Terminalia ferdinandiana* Exell. against extended-spectrum β-lactamase producing pathogens (ESBLs), and methicillin-resistant *Staphylococcus aureus* (MRSA) [14,15,16]. Phytochemical analyses of the *Terminalia* spp. in those studies identified a variety of compounds, including several noteworthy flavonoids, which may contribute to their antimicrobial properties. Flavonoids represent one of the largest classes of small-molecule secondary metabolites, synthesized in various parts of the plant. Plants produce flavonoids in response to microbial infections, and these compounds have demonstrated potent antibacterial activity against a broad range of pathogens [17]. Flavonoids not only selectively target bacterial cells, but also inhibit virulence factors and other microbial threats, such as biofilm formation [17]. Certain flavonoids can reverse AMR and enhance the effectiveness of existing antibiotic treatments [17].

This study investigated the antimicrobial activity of the flavonoids apigenin, fisetin, genistein, gossypetin, hispidulin, isoorientin, isorhamnetin, kaempferol, luteolin, myricetin, orientin, quercetin, rutin, taxifolin, and vitexin. We focused on the Gram-positive GI pathogen *Bacillus cereus*, as well as the Gram-negative species *Shigella sonnei*, *Shigella flexneri*, *Salmonella typhimurium*, *Citrobacter freundii*, *Alcaligenes faecalis*, and *Aeromonas hydrophila*. These pathogens were chosen for screening due to their significant role in GI-related infections, and their frequent resistance to multiple antibiotic classes, including β-lactams. The antibacterial activity of these flavonoids was also evaluated in combination with conventional antibiotics including ciprofloxacin, chloramphenicol, erythromycin, gentamicin, and tetracycline to determine their ability to enhance antibiotic effectiveness, potentially enabling their repurposing for clinical use. Orientin produced synergistic interaction in combination with erythromycin and gentamicin against *S. sonnei* and was therefore also assessed for efflux pump inhibitory activity. The toxicity of the selected flavonoids was evaluated using a human dermal fibroblast (HDF) cell line assay.

## 2. Results

### 2.1. Antimicrobial Activity of Flavonoids

Liquid microdilution assays were performed to assess the antibacterial activity of the flavonoids by quantifying their MIC values (Table 1). Isoorientin and orientin were highly effective against *A. faecalis* and *B. cereus* (MIC values against both = 62.5 µg/mL, 140 µM). Fisetin exhibited good activity against *A. hydrophilia* (MIC = 125 µg/mL, 436 µM). Similarly, hispidulin demonstrated good activity against *C. freundii* and *A. faecalis* (MIC = 125 µg/mL, 416 µM). Rutin showed good activity against *S. flexneri*, *A. hydrophilia*, and *A. faecalis* (MIC = 125 µg/mL, 204 µM). Apigenin, genistein, gossypetin, isorhamnetin, kaempferol, luteolin, myricetin, quercetin and taxifolin failed to inhibit the growth of any GI strain tested in this study.

### 2.2. Fractional Inhibitory Concentration

Flavonoids that had a MIC value ≤ 125 µg/mL against *A. faecalis*, *A. hydrophilia*, *B. cereus*, *C. freundii*, *S. flexneri*, *S. sonnei*, and *S. typhimurium* were each combined individually with a panel of antibiotics to assess the flavonoid’s effect on the potency of the antibiotic in the combination. Isoorientin and orientin had MIC values ≤ 125 µg/mL (Table 1) and were hence selected for combinational studies. Various classes of interactions were identified among the tested combinations (Table 2). Two synergistic, eighteen additive, and thirty-one non-interactive effects were identified, whilst no antagonistic interactions were observed.

### 2.3. Isobologram Analysis

**Figure 1:**
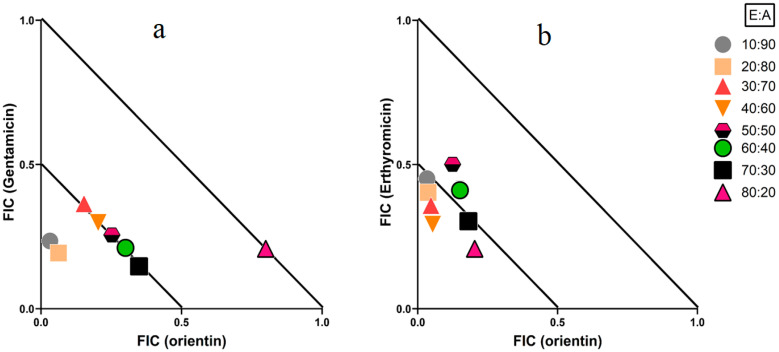
Isobolograms of varying ratios of (**a**) orientin and gentamicin against *S. sonnei*; (**b**) orientin and erythromycin against *S. sonnei*. Results are displayed as the mean MIC values of two independent experiments (*n* = 2). Ratio = % extract (E): % antibiotic (A). Ratios ≤ 0.5/0.5 represent synergy (∑FIC ≤ 0.5). Any ratios > 0.5/0.5 and ≤1/1 are considered additive (∑FIC > 0.5–1.0).

Orientin in combination with either gentamicin or erythromycin exhibited synergistic interactions against *S. sonnei* (Table 2). These combinations were further evaluated at different ratios and isobolograms were generated to determine the optimal ratio(s) for synergy. Only ratios demonstrating synergistic or additive effects were included in the isobologram analysis (Figure 1). Orientin in combination with gentamicin produced synergy at all ratios containing 10–70% orientin, whilst the ratio containing 80% orientin produced an additive effect (Figure 1a). Combining orientin with erythromycin produced synergistic interactions at ratios containing 10–40% and 70–80% orientin, whilst ratios containing 50–60% orientin produced additive effects (Figure 1b). Indifferent combinations do not enhance or diminish the antibacterial effects relative to the individual flavonoid or antibiotic components. Whilst these non-interactive combinations are considered safe for use, they offer no advantage over standalone therapies.

### 2.4. Accumulation and Efflux of Ethifium Bromide (EtBr)

**Figure 2:**
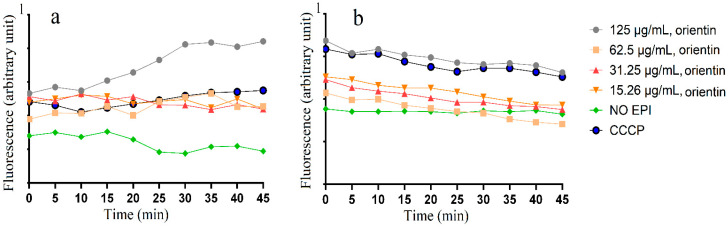
Effect of orientin in *S. sonnei* on (**a**) accumulation of EtBr; and (**b**) efflux of EtBr. CCCP was at half of the MIC value (15.62 µg/mL).

Orientin produced synergistic interaction against *S. sonnei* in combination with gentamicin and erythromycin. Therefore, the effects of orientin on EtBr accumulation and efflux were determined with *S. sonnei*. Four different concentrations of orientin (125 µg/mL, 62.50 µg/mL, 31.25 µg/mL, and 15. 26 µg/mL) were examined for EtBr accumulation and efflux activity (Figure 2). Bacterial cultures exposed to carbonyl cyanide 3-chlorophenylhydrazone (CCCP) were included as a positive control, whilst an untreated group without any efflux pump inhibitor (no EPI) served as a negative control. The no EPI control group (without EPI) showed the lowest level of EtBr accumulation (Figure 2a), indicating that these bacteria possess active efflux pump systems capable of expelling EtBr from the cell. Treatment with orientin at 125 µg/mL led to the highest accumulation of EtBr compared to other orientin concentrations and the positive control CCCP (Figure 2a). The inhibitory effect of orientin on the *S. sonnei* efflux was also assessed (Figure 2b). The negative control (no EPI) exhibited the lowest EtBr efflux inhibition activity. Notably, the CCCP positive control and 125 µg/mL orientin had a higher level of efflux pump inhibitory activity (Figure 2b). The results showed that orientin enhanced EtBr accumulation and reduced EtBr efflux compared to the negative control across most time points and concentrations, and these differences were statistically significant (*p* < 0.05) at most time points (Supplementary Tables S1 and S2).

### 2.5. Assessment of Toxicity

**Figure 3:**
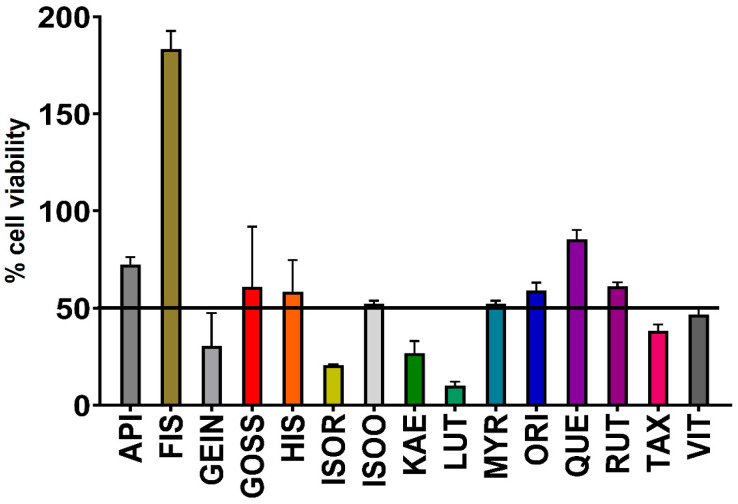
Flavonoid toxicity evaluation against HDF. API = apigenin, FIS = fisetin, GEIN = genistein, GOSS = gossyptein, HIS = hispidulin, ISOR = Isorhamnetin, ISOO = Isoorientin, KAE = kaempferol, LUT = luteolin, MYR = myricetin, ORI = orientin, QUE = quercetin, RUT = rutin, TAX = taxifolin, VIT = vitexin. % Cell viability is represented as three independent experiments, each with internal triplicates ± SEM (*n* = 9).

The toxicity of flavonoids was assessed using human dermal fibroblasts (HDF) cell viability assays, a standard model for evaluating the toxicity of flavonoids [18]. Cell viability of ≤50% was considered toxic, whilst >50% indicated non-toxicity. All flavonoids except genistein, isorhamnetin, kaempferol, luteolin, taxifolin, and vitexin were determined to lack toxicity at 300 µg/mL, demonstrating their apparent safety for therapeutic use (Figure 3). Interestingly, fisetin increased cell viability by more than 100%.

## 3. Discussion

**Figure 4:**
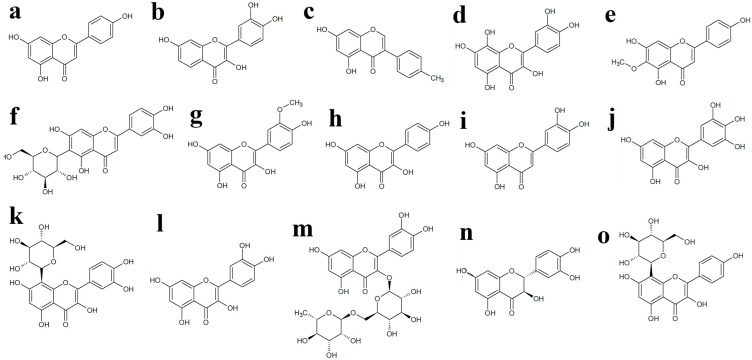
Chemical structure of (**a**) apigenin, (**b**) fisetin, (**c**) genistein, (**d**) gossypetin, (**e**) hispidulin, (**f**) isoorientin, (**g**) isorhamnetin, (**h**) kaempferol, (**i**) luteolin, (**j**) myricetin, (**k**) orientin, (**l**) quercetin, (**m**) rutin, (**n**) taxifolin and (**o**) vitexin.

Previously, our group reported the antibacterial activity of various *Terminalia* spp. against GI pathogens, and subsequent LC-MS studies highlighted numerous phytochemicals, including flavonoids, which may influence the antimicrobial activity of those *Terminalia* spp. [19]. Apigenin, genistein, gossyptein, isorhamnetin, kaempferol, luteolin, myricetin, quercetin, and taxifolin (Figure 4) failed to inhibit the growth of any bacterial strains tested in this study at a concentration of 250 µg/mL. Previous studies have reported the MIC of quercetin against *A. hydrophilia* to be 350 µg/mL in the disc diffusion assay [20], whilst the starting concentration of quercetin in our study was 250 µg/mL, which may account for the lack of observed inhibitory activity in our study. Also, in our study, we determined the antimicrobial activity of flavonoids using a liquid dilution assay which is regarded as more sensitive than the disc diffusion assay since it is not influenced by compound size or polarity [21]. Previous studies also reported the antibacterial activity of luteolin, rutin, and apigenin (Figure 4) against *E. faecalis,* whilst no activity was observed for quercetin and myricetin in agar disc diffusion assays [22]. However, the concentration at which the activity was screened was not documented in that study, making it challenging to compare with findings from other studies. Also, none of the earlier studies have investigated the antibacterial activity of the flavonoids screened in our study against the pathogens tested herein.

The antimicrobial properties of some flavonoids may operate through mechanisms distinct from those of conventional antibiotics, making them valuable candidates for enhancing antimicrobial therapies [23]. Flavonoids may exert an antimicrobial effect by targeting multiple cellular sites. One of their key molecular actions involves forming complexes with proteins through nonspecific interactions such as hydrophobic effects, hydrogen bonding, and covalent bond formation. As a result, their antibacterial activity may stem from their ability to inactivate enzymes, microbial adhesions, and cell envelope transport proteins. Myricetin, robinetin, and epigallocatechin (Figure 4) have been shown to inhibit DNA synthesis in several bacteria, including *S. aureus* and *Proteus vulgaris* [24]. The authors of the study proposed that the B ring of flavonoids may be involved in intercalation or hydrogen bonding with nucleic acid base stacking, potentially accounting for their inhibitory effect on DNA [24]. Another study examined the antimicrobial activity of fourteen different flavonoids against *E. coli* DNA gyrase in *S. aureus*, *S. epidermis*, *S. typhimurium*, *E. coli*, and *Strenotrophomonas maltophilia* [24]. The study revealed that enzyme inhibition was limited to flavonoids possessing hydroxylation on the B-ring [24]. Quercetin (Figure 4) has also been found to interact with the GyrB subunit of *E. coli* DNA gyrase and inhibit ATPase activity [25]. The binding of quercetin to the enzyme was confirmed by isolating *E. coli* DNA gyrase and measuring quercetin fluorescence, both in the presence and absence of the gyrase subunits [25].

Previously, a research team found that isophoraflavanone G possesses intensive antimicrobial activity against streptococci and MRSA [26]. The impact of sophoraflavanone G on membrane fluidity was examined using liposomal model membranes and compared to the less active flavanone, narigenin. At MIC equivalent concentrations, sophoraflavanone G significantly increased fluorescence polarization in the liposomes. This increase indicated changes in membrane fluidity within both hydrophobic and hydrophilic regions of the outer and inner membrane layers [26]. Epigallocatechin gallate, a highly antibacterial catechin found in green tea, was shown to cause leakage of small molecules from the intraliposomal space and disrupt bacterial membranes [27]. Catechins may disrupt lipid bilayers by directly penetrating them and compromising their barrier function or by inducing membrane fusion, leading to leakage of intramembranous contents and aggregation [27]. Licochalcone A and C, flavonoids derived from the roots of *Glycyrrhiza inflata* Batalin, inhibited the incorporation of radioactive precursors into macromolecules (RNA, DNA and protein) [28]. Licochalcones were also shown to interfere with energy metabolism in a manner similar to respiratory-inhibiting antibiotics, as energy is essential for the active uptake of various metabolites and the biosynthesis of macromolecules [28].

Synergistic combination therapies offer a promising approach in medical research for addressing AMR by enhancing the efficacy of existing antibiotics and reducing resistance development [12]. In this study, we observed two synergistic, eighteen additive, and thirty-one non-interactive interactions (Table 2). Synergistic interaction was observed when orientin was combined with either gentamicin or erythromycin. Orientin may block the resistance mechanism of gentamicin and erythromycin through mechanisms other than those to which these antibiotics have initially developed resistance, although this has not yet been verified, and further mechanistic studies are required. Gentamicin exerts its bactericidal effects by irreversibly binding to the 30S subunit of the bacterial ribosome, thereby inhibiting protein synthesis and leading to cell death [29]. Erythromycin binds to the 50S subunit of 70S ribosomes, inhibiting protein synthesis and thereby producing bactericidal effects [30]. Combinational antibiotic therapies may enhance the effectiveness of weak antimicrobials against bacterial infections. Rifampicin in combination with either quercetin or kaempferol produces synergistic interaction against MRSA isolates in vitro [31]. Quercetin and kaempferol alone produce 57.8% β-lactamase inhibition, although the inhibitory effect increased (75.8%) in combination with rifampicin [31]. The antimicrobial efficacy of ciprofloxacin, a fluoroquinolone derivative, was significantly enhanced by the addition of quercetin and kaempferol. Quinolones exert their antimicrobial effect by binding to topoisomerase IV in *S. aureus*, leading to DNA synthesis inhibition, extensive double-stranded DNA breaks, and growth arrest, ultimately causing cell death. Since quercetin and kaempferol also inhibit the catalytic activity of various bacterial topoisomerases, their synergistic interaction with ciprofloxacin may be attributed to this shared mechanism [32]. Importantly, the selected flavonoids were non-toxic in the HDF assay, with the exception of genistein, isorhamnetin, kaempferol, luteolin, taxifolin and vitexin (Figure 3). However, further validation of these flavonoids against additional cell lines and subsequent in vivo testing is required to confirm their safety for medicinal use.

In this study, we have also examined orientin’s efflux pump inhibitory activity. Notably, orientin produced synergistic effects against *S. sonnei* when tested in combination with either gentamicin or erythromycin. This highlights the potential of orientin as a candidate for further investigation into whether their synergistic activity was linked to efflux pump inhibition. We conducted EtBr efflux assays to assess efflux pump inhibitory activity. The use of EtBr as a marker for efflux pump inhibition is a well-established and widely used method [33]. EtBr intercalates with bacterial DNA, which can ultimately lead to cell death. To mitigate this, bacterial efflux proteins actively transport EtBr out of the cell [34]. Compounds that inhibit efflux pumps increase the intracellular retention of EtBr, antibiotics, and other harmful substances, thereby reducing bacterial survival [35]. Orientin enhanced the accumulation of EtBr (Figure 2a) and the inhibition of efflux from cells preloaded with EtBr (Figure 2b). This suggests that orientin acts as an efflux pump inhibitor in *S. sonnei*. Notably, the ethidium bromide accumulation activity of 125 µg/mL orientin in *S. sonnei* was statistically different to the negative control (No EPI) at 25 and 30 min compared to the negative control (Supplementary Table S1). In contrast, significant effects on ethidium bromide accumulation were only evident for CCCP after 45 min. Additionally, the effects of orientin on efflux pump inhibition activity were noted immediately and remained consistently increased compared to the untreated control for the duration of the assay (Supplementary Table S2). In contrast, 25 min was required for CCCP to significantly inhibit ethidium bromide efflux. To the best of our knowledge, no previous studies have examined the efflux pump inhibitory activity of the orientin against the pathogens tested in this study. Taken together, these results highlight the potential of orientin as an efflux pump inhibitor for use in antibiotic combination therapies.

Notably, several of the bacterial strains tested in our study were clinical isolates. These strains were selected as they have previously been reported to be resistant to several classes of clinical antibiotics [19,36]. Therefore, it was deemed that they would be appropriate for the studies presented herein, which focus on overcoming antibiotic-resistance mechanisms. However, future studies should also confirm this activity in well-characterized reference strains. Future studies should also examine the antimicrobial activity of flavonoids tested in this study against an extended panel of GI pathogens including other pathogenic bacteria, protozoa, fungi and viruses. Studies should also investigate whether the flavonoids studied in this study exhibit antibacterial study through other mechanisms, including (1) disruption of metabolic pathways, (2) interference with cell wall synthesis, (3) inhibition of protein synthesis, (4) impairment of membrane function, (5) interference with nucleic acid synthesis, and (6) inhibition of membrane function. Additionally, the toxicity evaluations reported herein screened the flavonoids against a single human cell line (HDF). Future studies are also required to confirm the safety of these compounds against a wider panel of relevant human cell lines. Additionally, whilst the flavonoids were tested for toxicity at 300 µg/mL, they were not screened across a range of concentrations, and therefore LC50 values were not determined. Future studies should also quantify the toxicity, which would allow for the calculation of therapeutic/safety indexes.

## 4. Materials and Methods

### 4.1. Materials

All solvents used in this study were of analytical grade (AR) and sourced from Ajax Fine-Chemicals Ltd., Taren Point, Australia. Flavonoids were obtained from multiple sources with their technical details listed in Table 3. Mueller–Hinton media was purchased from Oxoid Ltd., Thebarton, Australia. The components of the phosphate-buffered saline (PBS) were 0.0027 M potassium chloride, 0.01 M phosphate buffer and 0.137 M sodium chloride, pH 7.4. Unless otherwise specified, all additional chemicals and reagents were acquired from Sigma-Aldrich, Castle Hill, Australia.

### 4.2. Bacterial Strains

Reference strains of *Bacillus cereus* (ATCC 11778) and *Shigella sonnei* (ATCC 25931) were acquired from the American Tissue Culture Collection (ATCC). All other bacterial isolates tested in this study (*Alcaligenes faecalis*, *Aeromonas hydrophila*, *Citrobacter freundii*, *Salmonella typhimurium*, and *Shigella flexneri*) were kindly supplied by the teaching laboratories at Griffith University, Australia. The susceptibility and resistance of these bacterial strains to multiple antibiotics have previously been verified in our laboratory [14].

### 4.3. Bacterial Growth Conditions

Mueller–Hinton agar and broth powders were sourced from Oxoid Ltd. (Thebarton, Australia) and prepared according to the manufacturer’s guidelines. Initially, bacterial stock cultures were streaked on Mueller–Hinton agar plates and incubated at 37 °C for 24 h to obtain pure colonies. A single bacterial colony was then transferred to 50 mL of freshly prepared Mueller–Hinton broth and incubated at 37 °C until reaching the logarithmic growth phase, except for MRSA, which was incubated at 35 °C. The purity of each culture was confirmed by re-streaking onto Mueller–Hinton agar plates.

### 4.4. Antibacterial Susceptibility Assay

Standardized liquid-phase microdilution assay protocols were employed to assess the susceptibility of each bacterial strain to the flavonoids and control antibiotics [14]. All antibacterial susceptibility assays followed the recommendations of the Clinical and Laboratory Standards Institute (CSLI).

### 4.5. Analysis of Flavonoids: Antibiotic Optimal Ratios

The optimal ratios at which flavonoid and antibiotic produced synergistic interactions were assessed using standard methods [14]. Isobolograms were plotted to visualize the optimal/synergistic interactions between the flavonoid and the antibiotic.

### 4.6. Ethidium Bromide (EtBr) Accumulation Assay

This assay was conducted following standard protocols [37]. In summary, bacterial cultures were grown in Mueller–Hinton broth at 37 °C until reaching an optical density at 600 nm (OD_600_) of 0.8. The cultures were then centrifuged at 13,000 rpm for 3 min, the supernatant was discarded, and the pellet was washed and resuspended in phosphate-buffer saline (PBS). The optical density (OD) was adjusted to 0.4, and glucose (0.4% final concentration) along with EtBr (1 µg/mL final concentration) was added to 1 mL of bacterial suspension.

A total of 95 µL of the bacterial culture and 5 µL of orientin at four different concentrations (125 µg/mL, 62.5 µg/mL, 31.25 µg/mL, and 15.26 µg/mL) were dispensed into individual wells of a black bottom 96-well plate. PBS served as a negative control, while carbonyl cyanide 3-chlorophenylhydrazone (CCCP) at half its MIC concentration was used as a positive control. Fluorescence intensity (excitation: 530 nm, emission: 600 nm) was measured every 5 min for 45 min using a Molecular Devices SpectraMax M3 plate reader. All experiments were conducted twice, each with internal triplicates (*n* = 6), and results were expressed as mean ± SEM.

### 4.7. EtBr Efflux Assay

The impact of flavonoid test compounds on EtBr efflux activity was assessed using established methods [37]. Briefly, EtBr accumulation was monitored at 25 °C without glucose. EtBr was used at half of its MIC for bacterial strain to ensure optimal accumulation whilst preserving cell viability (Table 2). EtBr-loaded cells were centrifuged at 13,000 rpm for 3 min, then resuspended in PBS containing 0.4% glucose (but no EtBr). The OD_600_ was adjusted to 0.4 and 95 µL of the suspension was transferred to a black bottom 96-well plate, then 5 µL of orientin was added. Replica tubes without orientin served as negative controls, whilst cells treated with CCCP acted as a positive control. Fluorescence intensity (excitation: 530 nm, emission: 600 nm) was recorded every 5 min for a total of 45 min using a Molecular Devices SpectraMax M3 plate reader. Each experiment was conducted twice, each with internal triplicates (*n* = 6), and results were expressed as mean ± SEM.

### 4.8. Toxicity Studies

The safety of flavonoids was assessed by evaluating their toxicity in HDF cell assays using standard procedures [19]. The cell viability was calculated as a percentage of the untreated control using the following formula:

% cell viability = mean absorbance of flavonoid − mean absorbance of flavonoid blank/(mean absorbance of control − mean absorbance of blank)

Flavonoids with cell viability ≤ 50% were considered toxic, whereas those with viability > 50% were termed as non-toxic. Toxicity data were reported as the mean ± SEM from a minimum of three independent experiments, each conducted with internal triplicates (*n* = 9).

### 4.9. Statistical Analysis

One-way ANOVA was used to assess statistical significance between the control and treatment groups. Although statistical analysis could not be performed for the 96-well microtiter liquid dilution assays, the reliability of MIC values was ensured by conducting the assays three times on different days, with two replicates per assay (*n* = 6). This approach confirmed the reproducibility of all flavonoids, antibiotics, and their combinations.

## 5. Conclusions

AMR among GI pathogens has resulted in significant morbidity and mortality globally, highlighting the need to explore novel ways of developing new antibiotic treatments. In this study, we have examined the antibacterial activity of fifteen flavonoids against a panel of GI pathogens including Gram-positive and Gram-negative. Fisetin, hispidulin, isoorientin, orientin, rutin, and vitexin inhibited the activity of the tested pathogens. As these flavonoids were effective inhibitors of growth in bacterial strains that displayed resistance to several classes of antibiotics, it is possible that they exert their antimicrobial effects through novel and/or unstudied mechanisms. This remains to be determined in future studies. Orientin acts synergistically against *S. sonnei* in combination with either gentamicin or erythromycin. Its ability to inhibit efflux pumps was assessed and exhibited efflux pump inhibitory activity. Future studies should investigate whether the flavonoids tested in this study possess distinct antibacterial mechanisms and evaluate their potential as effective antibacterial agents. The toxicity of these compounds should be further evaluated using multiple cell lines to confirm their toxicity and safety for medicinal use.

## Figures and Tables

**Table 1:** MIC values (µg/mL, µM) of conventional antibiotics and flavonoids against the pathogens tested in this study.

	*C. freundii* µg/mL (µM)	*S. flexneri* µg/mL (µM)	*S. sonnei* µg/mL (µM)	*A. hydrophilia* µg/mL (µM)	*A. faecalis* µg/mL (µM)	*S. typhimurium* µg/mL (µM)	*B. cereus* µg/mL (µM)
Apigenin	-	-	-	-	-	-	-
Fisetin	250 (873)	250 (873)	-	125 (436)	250 (873)	250 (873)	-
Genistein	-	-	-	-	-	-	-
Gossypetin	-	-	-	-	-	-	-
Hispidulin	125 (416)	250 (832)	250 (832)	250 (832)	125 (416)	250 (832)	250 (832)
Isoorientin	125 (278)	125 (278)	125 (278)	125 (278)	**62.5** (140)	125 (278)	**62.5** (140)
Isorhamnetin	-	-	-	-	-	-	-
Kaempferol	-	-	-	-	-	-	-
Luteolin	-	-	-	-	-		-
Myricetin	-	-	-	-	-	-	-
Orientin	125 (278)	125 (278)	125 (278)	125 (278)	**62.5** (140)	125 (278)	**62.5** (140)
Quercetin	-	-	-	-	-	-	-
Rutin	250 (409)	125 (204)	250 (409)	125 (204)	125 (204)	250 (409)	250 (409)
Taxifolin	-	-	-	-	-	-	-
Vitexin	250 (578)	250 (578)	250 (578)	250 (578)	250 (578)	250 (578)	250 (578)
Positive control							
Tetracycline	0.63 (1.40)	0.63 (1.40)	0.63 (1.40)	0.63 (1.40)	-	1.25 (2.81)	0.63 (1.40)
Chloramphenicol	-	-	-	-	-	-	-
Ciprofloxacin	1.25 (3.77)	0.63 (1.88)	0.63 (1.88)	0.63 (1.88)	2.5 (7.55)	1.25 (3.77)	0.63 (1.88)
Gentamicin	0.31 (0.65)	0.31 (0.65)	0.31 (0.65)	0.07 (0.16)	0.31 (0.65)	0.63 (1.30)	0.31 (0.65)
Erythromycin	0.31 (0.42)	0.31 (0.42)	0.63 (0.84)	0.31 (0.42)	-	0.63 (0.84)	0.31 (0.42)
CCCP	7.81 (38)	7.81 (38)	15.62 (76)	3.90 (19)	15.62 (76)	7.81 (38)	3.90 (19)
EtBr	15.61 (40)	15.61 (40)	31.25 (80)	7.81 (19)	31.25 (80)	15.61 (40)	7.81 (19)
Negative control	-	-	-	-	-	-	-

MIC values of the flavonoids and antibiotic controls represent the mean values of three independent experiments (*n* = 3). MIC values are expressed as µg/mL and µM, - indicates no inhibition was observed at the tested concentration. Highly active MIC values for the flavonoid tests (<100 µg/mL) are highlighted in bold text.

**Table 2:** ∑FIC values for interactions between antibiotics and flavonoids.

		Tetracycline	Chloramphenicol	Ciprofloxacin	Gentamicin	Erythromycin
*A.faecalis*	Isoorientin	-	-	1.25	3	-
	Orientin	2	-	2	*0.63*	*0.63*
*A.hydrophilia*	Isoorientin	3	-	3	2.125	0.625
	Orientin	-	-	1.25	3	-
*B.cereus*	Isoorientin	4	-	4	*0.63*	*0.63*
	Orientin	4	-	4	*0.63*	*0.63*
*C. freundii*	Isoorientin	1.50	-	1	*0.63*	*0.63*
	Orientin	3	-	2	*0.63*	*0.63*
*S. flexneri*	Isoorientin	3	-	3	*0.63*	*0.63*
	Orientin	3	-	3	*0.63*	*0.63*
*S. sonnei*	Isoorientin	3	-	3	2.13	*0.63*
	Orientin	2.50	-	2.50	**0.50**	**0.25**
*S. typhimurium*	Isoorientin	2	-	2	*0.63*	*0.63*
	Orientin	4	-	4	*0.63*	*0.63*

∑FIC values of flavonoids in combination with conventional antibiotics against *A. faecalis*, *A. hydrophilia*, *B. cereus*, *C. freundii*, *S. flexneri*, *S. sonnei*, and *S. typhimurium*. **Synergy =≤ 0.5**; *additive => 0.5–1.0*; indifferent => 1.0–≤ 4.0; antagonistic => 4.0. FIC values were verified in duplicate experiments, - indicates no inhibition at any dose concentration tested.

**Table 3:** Information on the identity, source and purity of the flavonoids tested in this study.

Cat#	Flavonoid	Formula	Molecular Weight (g/mol)	Purity	Manufacturer
A12135	Vitexin	C_21_H_20_O_10_	432.4	>98%	Adooq Bioscience, Irving, TX, USA
18647	Taxifolin	C_15_H_12_O_7_	304.3	≥98%	Cayman Chemical, Ann Arbor, MI, USA
A10815	Rutin	C_27_H_30_O_16_	610.5	>98%	Adooq Bioscience
A10766	Quercetin	C_15_H_10_O_7_	302.2	>98%	Adooq Bioscience
A12096	Orientin	C_21_H_20_O_11_	448.38	>98%	Adooq Bioscience
A10615	Myricetin	C_15_H_10_O_8_	318.2	>98%	Adooq Bioscience
A10541	Luteolin	C_15_H_10_O_6_	286.2	>98%	Adooq Bioscience
A10495	Kaempferol	C_15_H_10_O_6_	286.2	>98%	Adooq Bioscience
16496	Isorhamnetin	C_16_H_12_O_7_	316.3	≥98%	Cayman Chemical
26862	Isoorientin	C_21_H_20_O_11_	448.4	≥95%	Cayman Chemical
A13945	Hispidulin	C_16_H_12_O_6_	300.26	>98%	Adooq Bioscience
G-500	Gossypetin	C_15_H_10_O_8_	318.24	>93%	Indofine Chemical, Hillsborough, NC, USA
10005167	Genistein	C_15_H_10_O_5_	270.2	≥98%	Cayman Chemical
A10388	Fisetin	C_15_H_10_O_6_	286.2	>98%	Adooq Bioscience
10010275	Apigenin	C_15_H_10_O_5_	270.2	≥98%	Cayman Chemical

## Data Availability

Data not presented within the manuscript is either available in the Supplementary Material section or is available from the corresponding author on reasonable request.

## Supplementary Materials

The following supporting information can be downloaded at: https://www.mdpi.com/article/10.3390/molecules30112300/s1, Table S1: Effect of different concentrations of orientin on the accumulation of ethidium bromide in *S. sonnei*; Table S2: Effect of different concentrations of orientin on the efflux of ethidium bromide in *S. sonnei*.
