# Supplementary material for: Flavonoids Identified in Terminalia spp. Inhibit Gastrointestinal Pathogens and Potentiate Conventional Antibiotics via Efflux Pump Inhibition
Source: Molecules. 2025 May 23;30(11):2300. doi: 10.3390/molecules30112300 (PMC12156397; doi:10.3390/molecules30112300)
Supplement: Supplementary file 1 [file molecules-30-02300-s001.zip › molecules-3643135-supplementary.pdf]

Supplementary data

Flavonoids identified in *Terminalia* spp. inhibit gastrointestinal pathogens and potentiate conventional antibiotic via efflux pump inhibition

Muhammad Jawad Yousaf Zai [1,2], Matthew James Cheesman [3], Ian Edwin Cock [1,2*]

[1] Centre for Planetary Health and Food Security, Griffith University, Brisbane, QLD 4111,

[2] School of Environment and Science, Griffith University, Brisbane, QLD 4111, Australia

[3] School of Pharmacy and Medical Sciences, Griffith University, Southport, QLD 4222, Australia

[*] Correspondence: I.Cock@griffith.edu.au; Tel.: +61737357637

Supp Table 1: Effect of different concentrations of orientin on the accumulation of ethidium bromide in *S. sonnei*

h:mm:ss	125 µg/mL		62.5 µg/mL		31.25 µg/mL		15.26 µg/mL		NO EPI	CCCP	
	Mean ± SEM	*p* value	Mean ± SEM	*p* value	Mean ± SEM	*p* value	Mean ± SEM	*p* value	Mean ± SEM	Mean ± SEM	*p* value
0:00:00	156987 ±381	0.13696	149176 ±206	0.96184	152940 ±2757	0.27945	156569 ±2534	0.11353	145655 ±1637	152996 ±1204	0.13837
0:05:00	157160 ±1331	0.15169	153823 ±3005	0.27761	156248 ±1832	0.21953	153199 ±1805	0.11353	143408 ±1625	151979 ±1166	0.13837
0:10:00	156634 ±812	0.15169	154987 ±4295	0.27761	153715 ±2743	0.27945	153665 ±2482	0.09642	145988 ±2285	153983 ±2899	0.07139
0:15:00	161599 ±1148	0.10824	153488 ±694	0.28751	153104 ±1566	0.27945	154281 ±1498	0.05568	144007 ±1269	154592 ±2236	0.08413
0:20:00	163855 ±986	0.08972	153253 ±3129	0.28751	157523 ±1847	0.20227	155399 ±1883	0.11353	145617 ±2655	156401 ±2883	0.08883
0:25:00	166200 ±380	**0.00553**	152979 ±1241	0.27761	154685 ±1454	0.14500	153019 ±1281	0.05568	137743 ±1460	155698 ±1130	0.05210
0:30:00	173470 ±2258	**0.04339**	156180 ±648	0.21649	153649 ±524	0.26977	153259 ±1520	0.07045	137545 ±1262	157099 ±1193	0.09303
0:35:00	175458 ±3714	0.09205	156214 ±385	0.28751	153485 ±1677	0.27945	153375 ±991	0.11353	144014 ±3253	157386 ±536	0.06548
0:40:00	172440 ±1932	0.08415	155444 ±2748	0.27761	152515 ±791	0.27945	156306 ±1321	0.05568	143129 ±2148	156965 ±141	0.04413
0:45:00	175312 ±3211	0.07092	151885 ±857	0.28751	153556 ±1582	0.20227	153331 ±1352	0.05442	138354 ±1114	158219 ±705	**0.03262**

Positive control = carbonyl cyanide 3-chlorophenylhydrazone (CCCP), Negative control = No efflux pump inhibitor (NO EPI), an experiment is twice, each with internal triplicates (*n*=6), and results presented as Mean ± SEM. *p* values highlighted in bold indicates results are statistically significant compared to the untreated control (NO EPI)

Supp Table 2: Effect of different concentrations of orientin on the efflux of ethidium bromide in *S. sonnei*

h:mm:ss	125 µg/mL		62.5 µg/mL		31.25 µg/mL		15.26 µg/mL		NO EPI	CCCP	
	Mean ± SEM	*p* value	Mean ± SEM	*p* value	Mean ± SEM	*p* value	Mean ± SEM	*p* value	Mean ± SEM	Mean ± SEM	*p* value
0:00:00	33207 ±523	**0.0168**	22169 ±760	0.26951	24576 ±397	0.06526	24069 ±1176	0.17986	17247 ±455	31372 ±383	**0.01754**
0:05:00	31366 ±564	**0.0199**	19361 ±441	0.55092	22256 ±436	0.15446	25189 ±603	0.09593	17537 ±482	31701 ±1202	0.05191
0:10:00	32817 ±1075	**0.0293**	18906 ±1077	0.84464	21352 ±523	0.21358	24048 ±800	0.13323	17557 ±487	32336 ±1656	0.06512
0:15:00	32892 ±2501	0.0590	18830 ±272	0.96368	22097 ±833	0.55391	23195 ±550	0.31839	18485 ±1338	28373 ±453	0.06802
0:20:00	29792 ±0.5	**0.01195**	17282 ±553	0.96368	21087 ±833	0.31489	22186 ±393	0.12034	17474 ±427	28382 ±820	0.05191
0:25:00	28207 ±378	**0.0168**	17996 ±924	0.79426	19077 ±167	0.17068	22291 ±602	0.12034	16288 ±398	26639 ±183	**0.01754**
0:30:00	27500 ±661	**0.0265**	16201 ±519	0.81169	18552 ±723	0.55473	19785 ±757	0.31839	17028 ±236	28154 ±880	0.05191
0:35:00	27628 ±790	0.0590	17107 ±1814	0.94170	18865 ±418	0.99387	18866 ±663	0.89317	18476 ±1415	27997 ±732	0.06802
0:40:00	28278 ±349	0.0588	14205 ±365	0.49982	18713 ±480	0.99387	19235 ±593	0.89317	18558 ±1250	26823 ±470	0.06802
0:45:00	25526 ±733	0.0588	13259 ±869	0.49982	17179 ±350	0.99387	18268 ±338	0.67449	17191 ±729	24558 ±664	0.06802

Positive control = carbonyl cyanide 3-chlorophenylhydrazone (CCCP), Negative control = No efflux pump inhibitor (NO EPI), an experiment is performed twice, each with internal triplicates (*n*=6), and results presented as Mean ± SEM. p values highlighted in bold indicates results are statistically significant compared to the untreated control (NO EPI)
